# Perovskite single-pixel detector for dual-color metasurface imaging recognition in complex environment

**DOI:** 10.1038/s41377-023-01311-2

**Published:** 2023-11-27

**Authors:** Jiahao Xiong, Zhi-Hong Zhang, Zile Li, Peixia Zheng, Jiaxin Li, Xuan Zhang, Zihan Gao, Zhipeng Wei, Guoxing Zheng, Shuang-Peng Wang, Hong-Chao Liu

**Affiliations:** 1 Institute of Applied Physics and Materials Engineering, University of Macau, Taipa, Macao SAR China; 2 State Key Laboratory of High Power Semiconductor Lasers, Changchun University of Science and Technology, Changchun, China; 3 Electronic Information School, and School of Microelectronics, Wuhan University, Wuhan, China; 4 Peng Cheng Laboratory, Shenzhen, China

**Keywords:** Metamaterials, Imaging and sensing

## Abstract

Highly efficient multi-dimensional data storage and extraction are two primary ends for the design and fabrication of emerging optical materials. Although metasurfaces show great potential in information storage due to their modulation for different degrees of freedom of light, a compact and efficient detector for relevant multi-dimensional data retrieval is still a challenge, especially in complex environments. Here, we demonstrate a multi-dimensional image storage and retrieval process by using a dual-color metasurface and a double-layer integrated perovskite single-pixel detector (DIP-SPD). Benefitting from the photoelectric response characteristics of the FAPbBr_2.4_I_0.6_ and FAPbI_3_ films and their stacked structure, our filter-free DIP-SPD can accurately reconstruct different colorful images stored in a metasurface within a single-round measurement, even in complex environments with scattering media or strong background noise. Our work not only provides a compact, filter-free, and noise-robust detector for colorful image extraction in a metasurface, but also paves the way for color imaging application of perovskite-like bandgap tunable materials.

## Abstract (web summary)

A compact, filter-free, double-layer perovskite single-pixel detector enables simultaneous recognition of overlapping dual-color metasurface images in complex environments.

## Graphical abstract

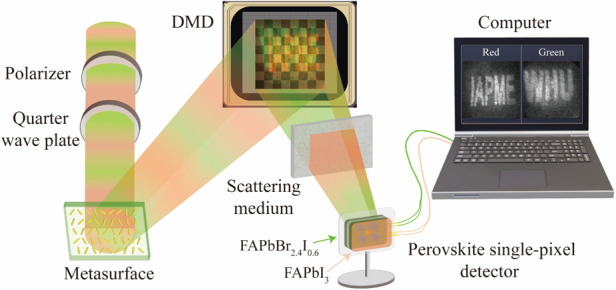

## Introduction

Data storage and extraction play a crucial role in information technology^1–3^. The information storage medium has undergone significant evolution, transitioning from paper to hard disks, with ever-increasing storage capacities and write-read speeds^4^. However, electrons used in electromagnetic materials, e.g., hard disks, possess quite limited degrees of freedom with only charge polarity and spin, which pose inherent constraints on their theoretical capacity. In contrast, optical materials with greater control of light degrees of freedom hold promise for larger information storage capacities^5–7^. A notable example is optical metasurfaces, which feature a periodic subwavelength unit cell structure, enabling independent control over several degrees of freedom of light^8–11^, including wavelength^12–14^, polarization^15,16^, amplitude^17,18^, momentum^19–21^, and phase^22–24^. This capability enables metasurfaces to achieve excellent optical multiplexing and makes them promising for optical image information storage with large capacities.

Although high-capacity image information can be stored in various degrees of freedom of light by strategically designing the geometry and material of the metasurface^25–28^, the extraction of the stored information is also a crucial end of optical information communication. At present, the extraction of optical image information is frequently carried out by employing silicon-based detectors, such as charge-coupled devices (CCDs) or complementary metal-oxide-semiconductor (CMOS)^29,30^. However, the ability of silicon-based detectors to fully extract optical information stored in different degrees of freedom of light is limited by the optical response properties of silicon materials. To achieve complete extraction of information, silicon-based detectors always require multiple measurements of the target with additional optical components. For instance, to extract the optical information stored at different wavelengths in metasurfaces, silicon-based detectors need to couple with filters. Similarly, for metasurface images with different polarization states, additional polarizers or waveplates are required to perform the detection. Moreover, traditional cameras based on CCD or CMOS chips rely on point-to-point imaging, which are sensitive to complex environments such as air turbulence and scattering media in the imaging path, resulting in degraded imaging results^31^.

To reduce the complexity of extracting information stored in different degrees of freedom and improve the environmental adaptability of detectors, researchers are searching for alternative optical materials to replace silicon-based detectors^32^. In the past few years, halide perovskite materials have demonstrated tremendous potential in the domains of photodetection and imaging^33^, owing to their low-cost solution preparation^34^, exceptional light absorption^35^, and tunable electronic bandgaps^36^. Detectors made of halide perovskite materials can be easily adjusted to respond to different wavelengths by modulating the components of the materials, which makes them a suitable option for developing sensors that are sensitive to different colors. However, perovskite materials are presently facing challenges in the preparation of highly integrated array detectors^37^. Very recently, perovskite materials have been proposed to combine with single-pixel imaging techniques to achieve multifunctional single-pixel detectors^38–42^ for color imaging^38,39,43^, wide-angle imaging^40^, and wavelength resolution of color targets^41,42^. Therefore, the excellent optoelectronic properties of perovskite single-pixel detectors inspire the extraction of the image information stored in metasurfaces with different degrees of freedom.

**Fig. 1:**
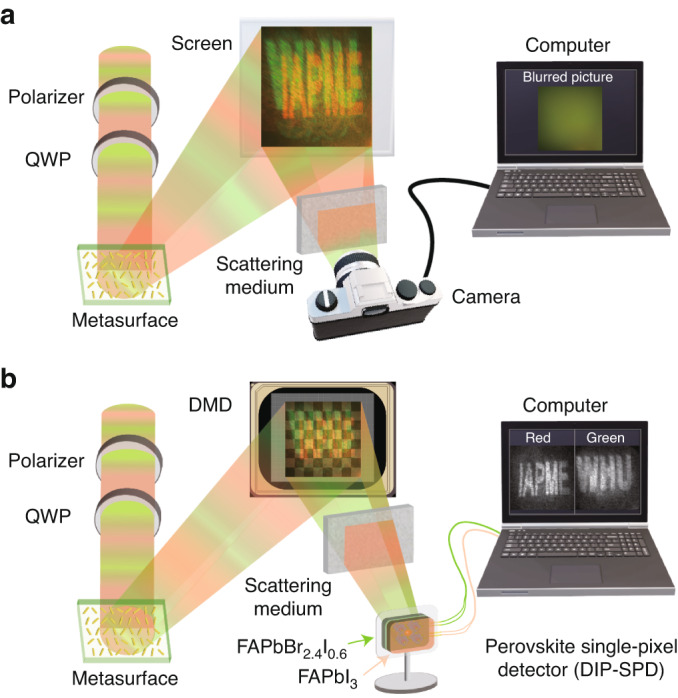
Comparison of imaging results between DIP-SPD and commercial cameras in complex environments. **a** Schematic diagram of the commercial camera imaging of the superimposed dual-color images reconstructed by the metasurface in the presence of a scattering medium. **b** Schematic diagram of the DIP-SPD single-pixel imaging of the superimposed dual-color images reconstructed by the metasurface in the presence of a scattering medium. QWP quarter-wave plate, DMD digital micromirror device

Different from a conventional camera shown in Fig. 1a, in this work, we design and fabricate a double-layer integrated perovskite single-pixel detector (DIP-SPD) that is utilized to recognize superimposed dual-color metasurface images in complex environments, as depicted in Fig. 1b. The top layer of our DIP-SPD is composed of a FAPbBr_2.4_I_0.6_ perovskite film, which responds to signals of the green image, while the bottom layer is made of a FAPbI_3_ film that detects signals of other wavelengths. The FAPbBr_2.4_I_0.6_ layer can act as a self-filtering element while reading the signal of the green image, which promises the identification and imaging of dual-color images in a single round measurement. Experimental results demonstrate that the DIP-SPD is capable of distinguishing two superimposed metasurface reconstruction images of different colors in a single round detection, even in complex environments with scattering media and background light, which is failed for a commercial CMOS camera as shown in Fig. 1a. Our work presents a compact, filter-free, and noise-robust perovskite detector for colorful images extraction in a metasurface. This not only provides a new inspiration for extracting images stored in other degrees of freedom of light, but also paves the way for color imaging of perovskite detectors.

## Results

### Photoelectric properties of DIP-SPD

**Fig. 2:**
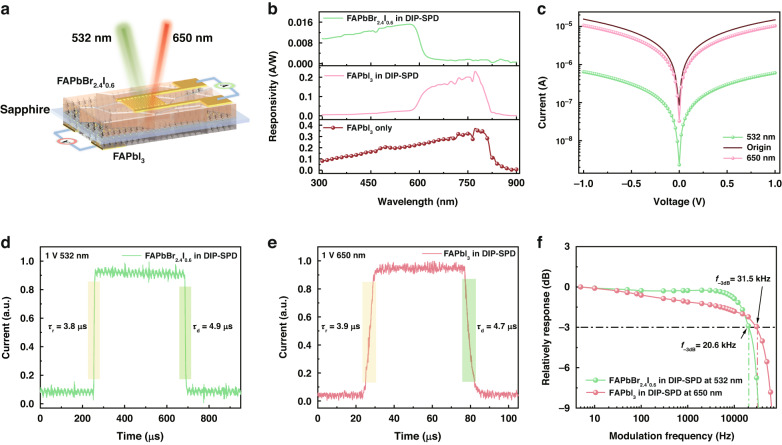
Structural schematic and photoelectric performance characterization of FAPbBr_2.4_I_0.6_ and FAPbI_3_ DIP-SPD. **a** Schematic diagram of the structure of DIP-SPD, with the FAPbBr_2.4_I_0.6_ device on top and the FAPbI_3_ device on the bottom. **b** The spectral responsivity of FAPbBr_2.4_I_0.6_ and FAPbI_3_ devices. **c** Current–voltage response curves of the FAPbI_3_ device to 532 nm and 650 nm lasers. **d**, **e** The photocurrent response times of FAPbBr_2.4_I_0.6_ and FAPbI_3_ devices at 1 V bias, respectively. **f** Responses of FAPbBr_2.4_I_0.6_ and FAPbI_3_ devices to the light modulation frequency

The DIP-SPD has a double-layer structure with two different perovskite devices which contain FAPbBr_2.4_I_0.6_ and FAPbI_3_ films, respectively, allowing it to differentiate between signals from images of different colors (Fig. 2a). The FAPbBr_2.4_I_0.6_ film is deposited on the top of the sapphire substrate to capture the modulated signal of the green color and permit the transmission of residual signal with longer wavelengths. The long wavelength signal (i.e., the red color image) is subsequently absorbed by the underlying FAPbI_3_ layer. Signals with different colors are output through the gold-interdigitated electrodes on both the top and bottom layers, which are utilized for image reconstruction in subsequent single-pixel imaging (SPI).

Figure 2b provides a visual representation of the devices’ response to different spectral ranges. The spectral responsivity curve of the FAPbI_3_ device (brown circular dots) has a flat spectral response in the wavelength range of 300–825 nm. However, due to the high absorption rate of the FAPbBr_2.4_I_0.6_ between 300 and 600 nm, which is coated on the top of the DIP-SPD, the spectral response range of the FAPbBr_2.4_I_0.6_ device in the DIP-SPD is limited to 300–600 nm. Meanwhile, the spectral response range of the FAPbI_3_ device is reduced and limited to 600–825 nm. The alteration of the spectral response range demonstrates the DIP-SPD’s effectiveness in distinguishing between signals of different colors.

The current-voltage curves of the FAPbI_3_ device (Fig. 2c) demonstrate that the photocurrent of the FAPbI_3_ device increases linearly with the applied bias within the voltage range of ±1 V. This manifests a typical ohmic contact between the gold electrode and the thin film, along with a photoconductive type detector. When subjected to the same 650 nm laser irradiation (98.1 mW/cm^2^), the photocurrent of the FAPbI_3_ device in the DIP-SPD (red line) closely resembles that of the single FAPbI_3_ device (brown solid curve), showing that the FAPbBr_2.4_I_0.6_ device has a negligible impact on the 650 nm incident signal. However, in contrast, the photocurrent generated by the FAPbI_3_ device in the DIP-SPD under 532 nm radiation (green line), with a power density of 112.6 mW/cm^2^, is observed to be one order of magnitude lower than that of the single FAPbI_3_ device. The observation suggests that the top FAPbBr_2.4_I_0.6_ device in DIP-SPD efficiently absorbs the 532 nm laser and operates as a self-filtering layer. The negligible attenuation of the 650 nm laser and strong absorption of the 532 nm laser by the FAPbBr_2.4_I_0.6_ device allows the DIP-SPD design to effectively recognize overlapping dual-color metasurface images.

As shown in Fig. 2d, e, both the top FAPbBr_2.4_I_0.6_ device and bottom FAPbI_3_ device of the DIP-SPD exhibit a rapid response to their respective wavebands. The rise times (i.e., the time for photocurrent to rise from 10 to 90% of the peak value) are approximately 4 *μ*s, while the drop times (i.e., the time for photocurrent to decrease from 90 to 10% of the peak value) are about 5 *μ*s. According to the light modulation frequency responsiveness data in Fig. 2f, the 3 dB bandwidths of the DIP-SPD for green and red signals are 20.6 kHz and 31.5 kHz, respectively. This provides further evidence of the rapid response capability of the DIP-SPD. Also, the DIP-SPD exhibits a high sensitivity in detecting weak light and demonstrates a wide linear-dynamic range of about 120 dB, along with good stability (Fig. S4). The comprehensive performance of the device enables it to be suitable for extracting variable optical information in complex environments.

### Recognition of superimposed dual-color metasurface images with DIP-SPD in single-pixel imaging

**Fig. 3:**
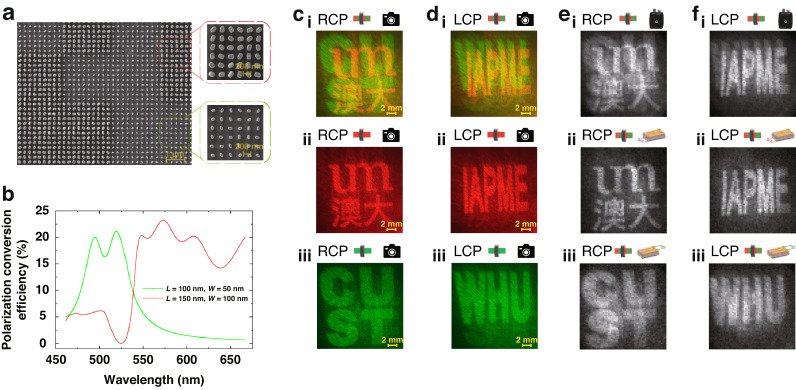
Imaging results with different incident lights and detectors. **a** The scanning electron microscopy (SEM) image of the metasurface. **b** The polarization conversion efficiency of two different sizes of silicon nanobricks in the metasurface for varying laser wavelengths. **c** (i) and **d** (i) The imaging result of the commercial camera when RCP and LCP laser beams of 612 nm and 532 nm are used as the light source. **c** (ii) and **c** (iii) (**d** (ii) and **d** (iii)) Imaging results of the commercial camera employing the laser beams with RCP (LCP) in 612 nm and 532 nm, respectively. **e** (i) (**f** (i)) The single-pixel imaging result of the commercial silicon SPD under an RCP (LCP) laser beam with both 612 nm and 532 nm. **e** (ii) and **e** (iii) (**f** (ii) and **f** (iii)) The imaging results corresponding to different colors extracted by the DIP-SPD simultaneously when an RCP (LCP) laser beam with both 612 nm and 532 nm is used as the light source

To confirm the ability of the DIP-SPD to simultaneously extract corresponding target images from different wavelengths in a single round measurement, a metasurface with both wavelength and polarization degrees of freedom was designed (Section 1 of the Supporting Information describes the design and fabrication process for the metasurface). The metasurface consists of silicon nanobricks with different sizes and special phase arrangements that enable them to respond to lasers of different wavelengths and polarization. The scanning electron microscope (SEM) image of the metasurface shown in Fig. 3a displays the arrangement of silicon nanobricks with two different sizes, i.e., 150 nm × 100 nm (*L* = 150 nm, *W* = 100 nm) and 100 nm × 50 nm (*L* = 100 nm, *W* = 50 nm). The dashed boxes in Fig. 3a depict the nanobricks distribution in higher magnification for the red and green images. Figure 3b depicts the polarization conversion efficiency of the metasurface target in different wavelengths. The nanobrick array with the size of *L* = 100 nm and *W* = 50 nm exhibits a relatively high polarization conversion efficiency in the wavelength range of 475–535 nm with the average value exceeding 16%. However, for lasers with longer wavelengths, the efficiency rapidly decreases to below 5%. In contrast, the nanobrick array with the size of *L* = 150 nm and *W* = 100 nm exhibits relatively high polarization conversion efficiency in the wavelength range of 535–650 nm with the average value exceeding 18%, but for shorter wavelengths, the efficiency decreases to less than 5%. The metasurface can reconstruct different images by changing the wavelength and polarization of the incident laser. Specifically, using a left-handed circularly polarized (LCP) laser beam in 535–650 nm (or 475–535 nm) as the light source results in the reconstruction of the “IAPME” (or “WHU”) image, while using a right-handed circularly polarized (RCP) laser beam in 535–650 nm (or 475–535 nm) results in the reconstruction of the “UM” (or “CUST”) image. When a mixed laser beam containing multiple wavelengths is used as the source, the image reconstructed by the metasurface is a superposition of the images from different colors.

To verify the ability of the DIP-SPD to extract images stored in a metasurface with different wavelengths, a commercial CMOS camera (Sony IMX766) and a commercial silicon detector (Thorlabs PDA100A2) are selected for comparisons. During the experiment, a supercontinuum laser (NKT Photonics SuperK EXTREME) is utilized as the light source. The center wavelength for the red image was 612 nm with a bandwidth of ±2 nm, while the center wavelength for the green image was 532 nm with a bandwidth of ±5 nm, resulting in a total power of approximately 3 mW for both images. With a traditional commercial CMOS camera, the reconstructed metasurface images projected onto a screen are captured and shown in Fig. 3c, d. Figure 3c (i) presents a superimposed image (“UM” and “CUST”) when the metasurface is illuminated by an RCP beam with both 612 nm and 532 nm, indicating that the commercial CMOS camera cannot distinguish image information in different wavelengths simultaneously. Figure 3c (ii) and (iii) illustrate that the commercial camera can accurately acquire the corresponding image only when the metasurface is incident separately by RCP beams of different wavelengths. A similar result is shown in Fig. 3d (i) with an incident dual-color LCP beam, where a superimposed image (“IAPME” and “WHU”) is read out by a traditional camera. The corresponding monochrome images captured by the traditional camera are exhibited in Fig. 3d (ii) and (iii).

Different from the traditional imaging process, single-pixel imaging with a commercial silicon SPD or the DIP-SPD involves the modulation of images reconstructed by the metasurface with a series of speckle patterns displayed on the DMD (X-digit F4100). The modulation patterns ${H}_{i}(x,y)$ displayed on the DMD are generated by a 64-order Hadamard matrix (several images of Hadamard patterns are presented in Fig. S5 of the Supporting Information). When using an RCP (LCP) laser beam with wavelengths of 612 nm and 532 nm as light sources, the commercial silicon SPD is only able to output one set of overlapped signals, resulting a superimposed image shown in Fig. 3e (i) (Fig. 3f (i)). In contrast, the DIP-SPD is capable of distinguishing image information of different colors, resulting in the output of two distinct image signals: one for the red image (${b}_{i}^{r}$) and the other for the green image (${b}_{i}^{g}$). By correlating the two distinct signals ${b}_{i}^{r}$ and ${b}_{i}^{g}$ with the corresponding modulation patterns ${H}_{i}(x,y)$ respectively, the red image ${T}^{r}$ and green image ${T}^{g}$ can be extracted from the overlapping dual-color images. The specific expression of the differential correlation imaging algorithm employed is presented as follows^44^:

1 $${T}^{j}\left(x,y\right)=\frac{1}{M}\mathop{\sum }\limits_{i=1}^{M}{{\rm{b}}}_{i}^{j}{H}_{i}\left(x,y\right)-\frac{\bar{b}}{\bar{h}}\frac{1}{M}\mathop{\sum }\limits_{i=1}^{M}{h}_{i}{H}_{i}\left(x,y\right)$$

where $j=r$ or $g$, representing the red or green images of the metasurface. $\bar{b}=\frac{1}{M}\mathop{\sum }\nolimits_{i=1}^{M}{b}_{i}$ denotes the average value of the image signals. $M=8192$ is the total number of the modulation patterns. $\bar{h}=\frac{1}{M}\mathop{\sum }\nolimits_{i=1}^{M}{h}_{i}$ represents the average value of the modulation pattern signal, where ${h}_{i}=\mathop{\sum }\nolimits_{x=1}^{n}\mathop{\sum }\nolimits_{y=1}^{n}{H}_{i}\left(x,y\right)$ signifies the signal intensity of each modulation pattern.

By setting the modulation frequency of the DMD to 1000 Hz and using a laser with both 612 nm and 532 nm as the light source, SPI results can be obtained as shown in Fig. 3e, f (Figure S8 in the Supporting Information depicts the imaging results of DIP-SPD at different DMD modulation frequencies ranging from 1000 to 18000 Hz). Similar to the results with a traditional camera in Fig. 3c (i), d (i), the commercial silicon SPD is unable to simultaneously identify images of different colors from superimposed dual-color metasurface images, as shown in Fig. 3e (i) and f (i), even though they incorporate the SPI method.

In contrast, the DIP-SPD can accurately identify images of different colors from the superimposed dual-color metasurface images in a single-round measurement. Figure 3e (ii) and (iii) display the different color images obtained simultaneously by the DIP-SPD in a single extraction process using an RCP laser beam with both 612 nm and 532 nm as the light source. Figure 3e (ii) shows the extracted image from the bottom FAPbI_3_ device in the DIP-SPD, while Fig. 3e (iii) depicts the extracted image from the top FAPbBr_2.4_I_0.6_ device. Similarly, Fig. 3f (ii) and (iii) show the images extracted by the DIP-SPD in a single round measurement using an LCP laser beam with both 612 nm and 532 nm as the light source, respectively. In Section 3.2 of the Supporting Information, we evaluate the peak signal-to-noise ratio (*P*_*SNR*_) of the reconstructed image of the bottom FAPbI_3_ layer through simulation experiments. The result indicates that the residual signal of the green image has a negligible effect on the quality of the reconstructed image of the bottom FAPbI_3_ layer. Compared with the imaging results of the traditional camera and the commercial silicon SPD, it can be observed that only the DIP-SPD is capable of simultaneously extracting image information stored in different wavelengths in a single round measurement, with nearly the same imaging quality.

### Imaging capabilities of DIP-SPD in complex environments

**Fig. 4:**
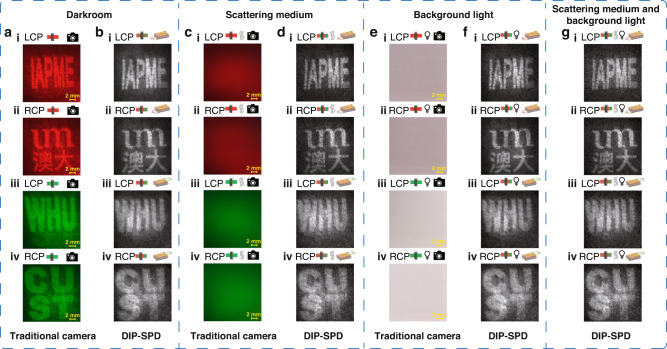
Comparison of imaging results between the traditional commercial camera and the DIP-SPD in complex environments. The imaging results of **a** the traditional camera (**b** the DIP-SPD) in a dark environment without scattering media. The imaging results of **c** the traditional camera (**d** the DIP-SPD) in a dark environment with a scattering medium. The imaging results of **e** the traditional camera (**f** the DIP-SPD) in an environment with background light. **g** The imaging results of the DIP-SPD in an environment where both the scattering medium and background light exist simultaneously

Compared to the traditional camera, the DIP-SPD also enables the effective extraction of image information in complex imaging environments with scattering media or background light. Figure 4 exhibits the imaging results of the DIP-SPD and the traditional camera on the overlapping dual-color metasurface images under three different conditions: darkroom, scattering medium, and background light. The darkroom environment is achieved by blocking out surrounding lights, and the illumination of metasurface images at the camera or detector location in this environment is 18 lux. For the case of scattering medium, a 100 μm thick laminating pouch film from Miracle company is utilized to serve as the scattering medium in front of the cameras as shown in Fig. 1 (Figure S10 of the Supporting Information displays the microscopy image of the scattering medium). For the case with background light, a desk lamp (Opple MT-HY03T-222) is employed to generate additional illumination (~1477 lux) at the camera or detector, which is around 100 times brighter than the metasurface images needed to be extracted.

Figure 4a, c depict the imaging results of the traditional commercial camera in the dark environment without and with the scattering medium. It is evident that the presence of the scattering medium significantly degrades the imaging quality, and even the outline of the image cannot be visualized. The image deterioration occurs since the point-to-point mapping of the traditional camera to the target is disrupted by the scattering effect of the wavefront (Figure S11 of the Supporting Information presents the imaging results of the traditional commercial camera when half of the target image is obscured by the scattering medium). Compared with the imaging results of the traditional camera, the DIP-SPD is capable of extracting image information of different colors simultaneously from the superimposed dual-color metasurface images even in the presence of a scattering medium, as illustrated in Fig. 4d. Moreover, the imaging quality of the results in Fig. 4d is nearly identical to that captured in a darkroom case shown in Fig. 4b, manifesting the robustness of DIP-SPD in scattering media. In addition to laminating pouch film, our single-pixel extraction systems can reconstruct clear images through other slow-changing scattering media based on its pattern modulation and bucket detection method (Detailed discussion in Section 3.6 of the Supporting Information).

Metasurface imaging always requires a coherent laser beam for its illumination, where the laser power is of great importance for the demonstration of metasurface images. A laser with low power will prevent the clear and visible observation of metasurface images in a daily environment with strong background light, whereas a high-power laser will accumulate lots of heat and increase the damage probability of the metasurface. We further test different detectors in the complex environment with background light. Due to the low light intensity of the images reconstructed by the metasurface (~18 lux), the images captured by the traditional camera are overwhelmed by the strong background light (~1477 lux). Figure 4e depicts the imaging results of the commercial camera with a milky and blurred appearance, which hinders the correct extraction of image features. Unlike the commercial camera, the DIP-SPD enables effective recognition of image information in the environment with background light, as shown in Fig. 4f. The resolution and clarity of the results in Fig. 4b, f are comparable, indicating the robustness of DIP-SPD to background light. The remarkable imaging results of DIP-SPD can be attributed to the wavelength selectivity and high detection sensitivity of the perovskite devices. Moreover, the imaging capability of the DIP-SPD is also verified in complex environments with both scattering media and background light, as shown in Fig. 4g. The precise imaging results presented in Fig. 4g demonstrate the effectiveness of the DIP-SPD in extracting image information stored at different wavelengths, even in the presence of multiple noises at the same time. More analyses of DIP-SPD detection signals in background light cases can be found in Fig. S13 of the Supporting Information.

## Discussion

Compared with conventional proposals that utilize silicon-based commercial cameras for extracting information stored at different wavelengths in metasurfaces^29,30^, our DIP-SPD can clearly extract dual-color images in a complex environment with scattering media and background light. Different from the image extraction schemes that use a single-pixel perovskite detector for point scanning, our DIP-SPD system circumvents the requirement for an *x*-*y* biaxial moving platform, enabling large-size image extraction^45–48^. The single-pixel detector in our DIP-SPD system does not need to be moved, and the object can be imaged in seconds by the fast-operating DMD, which is much faster than the traditional raster scanning single-pixel imaging method (~1 h)^49^. Compared with the perovskite SPI detector for colorful imaging^38^, our DIP-SPD demonstrated its advantage as a compact filter-free detector that can extract complete image information stored at different wavelengths in a single round measurement.

In summary, we design and fabricate a double-layer integrated single-pixel detector that utilizes FAPbBr_2.4_I_0.6_ and FAPbI_3_ perovskite thin films, and demonstrate its capability to recognize overlapping dual-color metasurface images in complex environments by following a SPI modality. The ability of the DIP-SPD to simultaneously extract images stored at different wavelengths stems from its double-layer stacking structure: a FAPbBr_2.4_I_0.6_ film is utilized on the top layer to enable the response and self-filtering function for the wavelength range of 300–600 nm, while a FAPbI_3_ film is placed on the bottom layer to output the response signal in the 600–820 nm band. These two films work in tandem to enable the extraction of two distinct color metasurface images in a single round measurement. In addition, the 5 μs response speed of both the FAPbBr_2.4_I_0.6_ and FAPbI_3_ films makes the DIP-SPD well-suited for the application of fast SPI imaging systems. More importantly, the DIP-SPD SPI system can also accurately identify superimposed dual-color metasurface images in complex environments with scattering media and background light. Targeting metasurface image extraction with different wavelengths, our work overcomes the challenge faced by traditional CMOS cameras and commercial silicon-based SPDs, which require additional wavelength filters and multiple round measurements. Through further refinement of the perovskite film composition, it is possible to obtain a full-color image of the target object utilizing a single-pixel detector featuring a three-layer structure. Three different layers can capture the image of red, green, and blue colors, respectively. This work not only provides ideas for the extraction of metasurface images stored in different degrees of freedom of light, but also paves the way for color imaging applications of perovskite materials.

## Materials and methods

### Materials

Lead iodide (PbI_2_ 99.99%), Formamidine iodide (FAI, 99.99%), Lead bromine (PbBr_2_ 99.99%), rubidium chloride (RbCl, 99.99%), and methylammonium chloride (MACl) were purchased from Xi’an Polymer Light Technology Corp., China. N,N-dimethylformamide (DMF, anhydrous, 99.9%), dimethyl sulfoxide (DMSO, anhydrous, 99.9%), toluene (anhydrous, 99.8%), and isopropanol (IPA) were received from Sigma Aldrich (USA). Unless otherwise stated, the reagents and solvents were used directly without any purification.

### Preparation of the DIP-SPD

Double-polished sapphire substrates were first cleaned with detergent and deionized water, then with acetone and IPA, followed by ultraviolet ozone treatment (~15 min). (1) The bottom device was prepared using a two-step spin coating method. Firstly, 1.5 M of PbI_2_ in DMF and DMSO (9:1) solution with added 0.075 M RbCl was spin-coated on sapphire at 3000 rpm for 30 s and annealed at 70 °C for 2 min in the nitrogen glove box. Then, for FAPbI_3_ perovskite film deposition, a salt solution (50 *μ*L) of FAI: MACl (90 mg: 15 mg in 1 mL IPA) was spin-coated onto the PbI_2_ substrate at a speed of 2500 rpm for 30 s, and the film was annealed in air (< 30% humidity) at 150 °C for 15 min and in a nitrogen glove box at 100 °C for 10 min. The Au electrodes were then prepared using fabricated using the interdigital electrode masks by thermal vapor deposition and the device was encapsulated in a nitrogen atmosphere. (2) The top device was prepared on the back side of the substrate in a one-step anti-solvent process. 1.5: 1.05: 0.45 FABr, PbBr_2_, and PbI_2_ were dissolved in 21: 4 mixed DMF and DMSO to form the precursor with a concentration of 1.5 M with added 5% M of RbCl. The precursor must be filtered with a 0.22 μm PTEE filter. To form the FAPbBr_2.4_I_0.6_ perovskite film, the precursor solution was spin-coasted at 4500 rpm for 60 s, and 500 mL toluene was added after 15 s. Then, the films were annealed in a nitrogen atmosphere at 80 °C for 5 min and later completed the encapsulation. Finally, Au interdigital electrodes were fabricated by the thermal evaporation method, sequentially applying physical masks on the surface of different films. The pitch and length of the fork interdigital electrodes were 80 μm and 3 mm, with 20 pairs of interdigital on a single electrode. As a result, the device has an effective area of 4.8 × 10^−2^ cm^2^.

### Characterization of the DIP-SPD

The high-precision electrical characteristics (*I*–*V*, *I*–*t*) were measured by a semiconductor analyzer (Keysight B1500A, USAZ) equipped with a probe station (Semishare) and a silver probe. The excitation light source of the device adopts the semiconductor laser at 532 nm (maximum power: 50.68 mW) and 650 nm (maximum power: 44.13 mW) and the light spot area of 0.45 cm^2^. The laser power was regulated by an attenuator (Thorlabs FW1) and measured by optical power meters (VEGA OPHIR PD300-UV), and the laser modulation signal was generated by a Function/arbitrary waveform generator (RIGOL DG4062, China). The high-resolution *I*-*t* (response time) signals were obtained from a Mixed Domain oscilloscope (MDO4054C, Tektronix, USA) with a preamplifier (SR570, Stanford Research Systems, USA). Noise current was measured at different frequencies (from 0.01 Hz to 1000 Hz) by a lock-in amplifier (Stanford Research System, SR830). The spectral responsivity was carried out with a response spectrometer (Zolix Instruments, China), a white xenon lamp source (150 W) was separated to produce light at continuous wavelengths using a monochromator, the lock-in amplifier collected the current signals, and the intensity of the light was calibrated by a commercial silicon detector (Thorlabs PDA100A2). All measurements were taken at room temperature in a nitrogen environment.

### Imaging process of DIP-SPD-based SPI

The schematic diagram of the DIP-SPD-based SPI is shown in Fig. 1b. Laser beams of different wavelengths were generated by a supercontinuum laser (NKT Photonics SuperK EXTREME). Before being incident onto the metasurface to obtain the metasurface images, the laser beams were first polarized by a polarizer, and then adjusted to suitable circular polarization via a quarter-wave plate (QWP). The metasurface reconstructed the target images under the laser beams, which were subsequently modulated by the DMD (X-digit F4100). The current signals corresponding to the different modulation patterns were outputted by the DIP-SPD and collected by a lock-in amplifier, then recorded on an oscilloscope. The recorded data were imported into MATLAB software, and the differential algorithm was employed to restore the images.

### Supplementary information

Perovskite single-pixel detector for dual-color metasurface imaging recognition in complex environment
