# Supplementary material for: Perovskite single-pixel detector for dual-color metasurface imaging recognition in complex environment
Source: Light Sci Appl. 2023 Nov 27;12:286. doi: 10.1038/s41377-023-01311-2 (PMC10679139; doi:10.1038/s41377-023-01311-2)
Supplement: Supplementary file 1 — Perovskite single-pixel detector for dual-color metasurface imaging recognition in complex environment [file 41377_2023_1311_MOESM1_ESM.docx]

# Supporting Information

# Perovskite single-pixel detector for dual-color metasurface imaging recognition in complex environment

Jiahao Xiong^1,#^, Zhi-Hong Zhang^1,2,#^, Zile Li^3,4,#^, Peixia Zheng^1^, Jiaxin Li^3^, Xuan Zhang^1^, Zihan Gao^1^, Zhipeng Wei^2^, Guoxing Zheng^3,4,^*, Shuang-Peng Wang^1,^* and Hong-Chao Liu^1,^*

^1^ Institute of Applied Physics and Materials Engineering, University of Macau, Tapia,

Macao SAR, 999078, China.

^2^ State Key Laboratory of High Power Semiconductor Lasers, Changchun University of Science and Technology, Changchun, 130022, China

^3^ Electronic Information School, and School of Microelectronics, Wuhan University, Wuhan, 430072, China

^4^ Peng Cheng Laboratory, Shenzhen, 518055, China

^#^ J. H. X., Z. H. Z., and Z. L. L. contributed equally to this work.

*E-mail: G. X. Z. (gxzheng@whu.edu.cn); S.-P. W. (spwang@um.edu.mo); H.-C. L. (hcliu@um.edu.mo)

This file includes:

Section 1. Design and fabrication of metasurface target in experiments

1-1 Design of metasurface target

1-2 Fabrication of metasurface target

Section 2. Photoelectric performance of FAPbBr_2.4_I_0.6_ and FAPbI_3_ DIP-SPD

Section 3. DIP-SPD single-pixel imaging system in complex environment

3.1 Hadamard patterns utilized in experiments

3.2 Simulation analysis of the reconstructed image quality of the bottom FAPbI_3_ layer

3.3 Single-pixel imaging results of DIP-SPD at different DMD modulation frequencies

3.4 Imaging results under irradiation with linearly polarized light of mixed wavelengths

3.5 Effect of the scattering medium on traditional camera imaging

3.6 Single-pixel signal in an environment with scattering media

3.7 Single-pixel detection signals in background light environment

## Section 1. Design and fabrication of metasurface target in experiments

### 1.1 Design of metasurface target


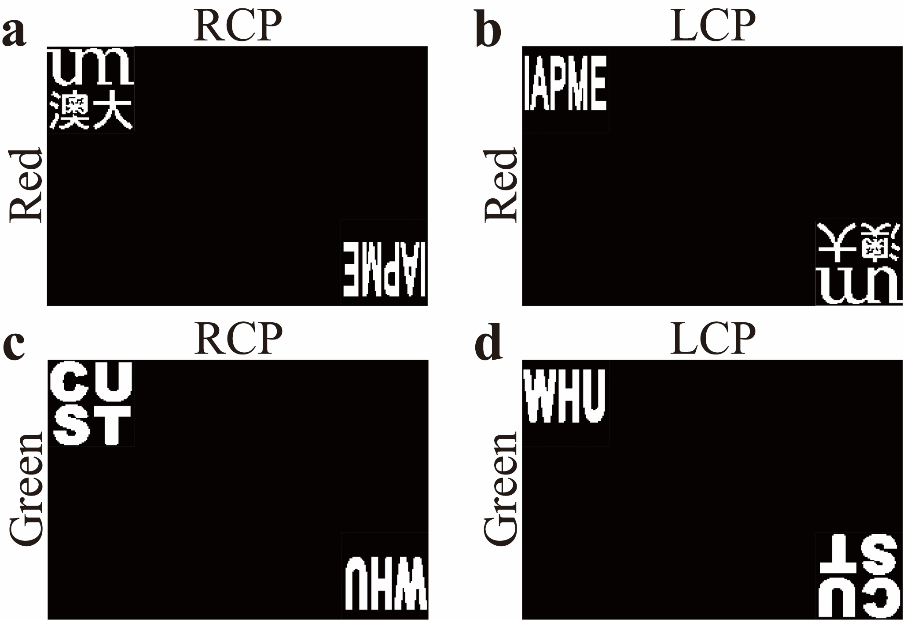


**Fig. S1 Predefined metasurface images were reflected under red or green lasers with right circular polarization (RCP) and left circular polarization (LCP).** **a** Preset reflection image of the metasurface under RCP red light illumination. **b** Preset reflection image of metasurface under LCP red light illumination. **c** Preset reflection image of metasurface under RCP green light illumination. **d** Preset reflection image of the preset metasurface under LCP green light illumination.

Figure S1 displays the designed reflected images of the metasurface when illuminated by lasers of varying colors and polarizations. The metasurface utilized in the experiment is fabricated through silicon processing on a sapphire substrate (SOS), which will reflect two sets of different images under the irradiation of lasers in the red and green bands, respectively. Figures S1(a) and S1(c) depict the images of “UM” and “IAPME” can be obtained under the irradiation of an RCP laser in the red band, while the images of “CUST” and “WHU” under the irradiation of RCP laser in the green band. Additionally, the reflected images from the metasurface exhibit polarization-dependent transformations, with the images reflected under left-handed circular polarization (LCP) being rotated 180° relative to those reflected under right-handed circular polarization (RCP), as illustrated in Figs. S1(b) and S1(d).

During the design process, the nanobrick structure was chosen to achieve the intended function. On the sapphire substrate, two crystalline silicon nanobricks were arranged, with dimensions of 150 nm × 100 nm (L = 150 nm, W = 100 nm) and 100 nm × 50 nm (L = 100 nm, W = 50 nm), respectively. These nanobricks were designed to respond to lasers in the red and green bands. The pitch of the metasurface cell was set to 300 nm with a height of 230 nm. Next, the phase distribution matrices, φ1 and φ2, were designed based on the holographic images at different wavelengths, as shown in Figs. S2(a) and S2(b). In the phase distribution matrix φ1, groups of adjacent 10 × 10 elements were selected and half of the groups were randomly replaced with the corresponding element values from the phase distribution matrix φ2. Figure S2(c) displays a partial view of the gds file used for processing the metasurface.


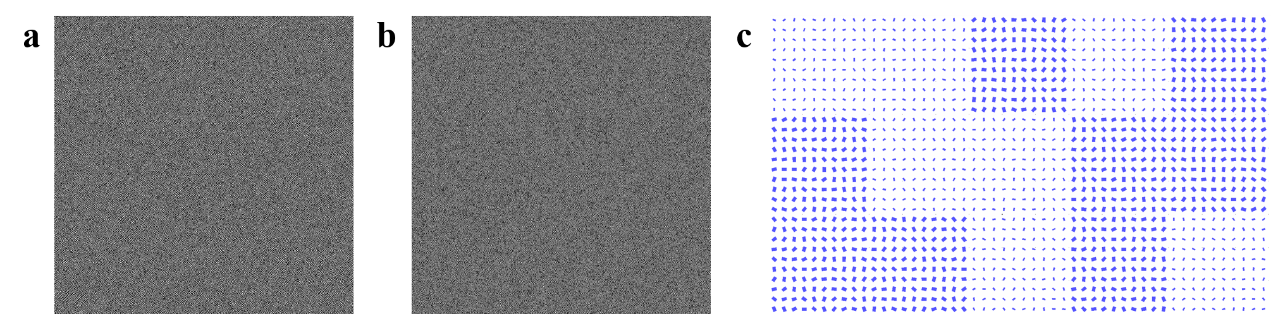


**Fig. S2 Phase distribution matrices of holographic images at different wavelengths and partial graphics of the gds file of the metasurface. a** The phase distribution matrix φ1 of the red image. **b** The phase distribution matrix φ2 of the green image. **c** Partial graphics of the gds file of the metasurface.

### 1.2 Fabrication of metasurface target


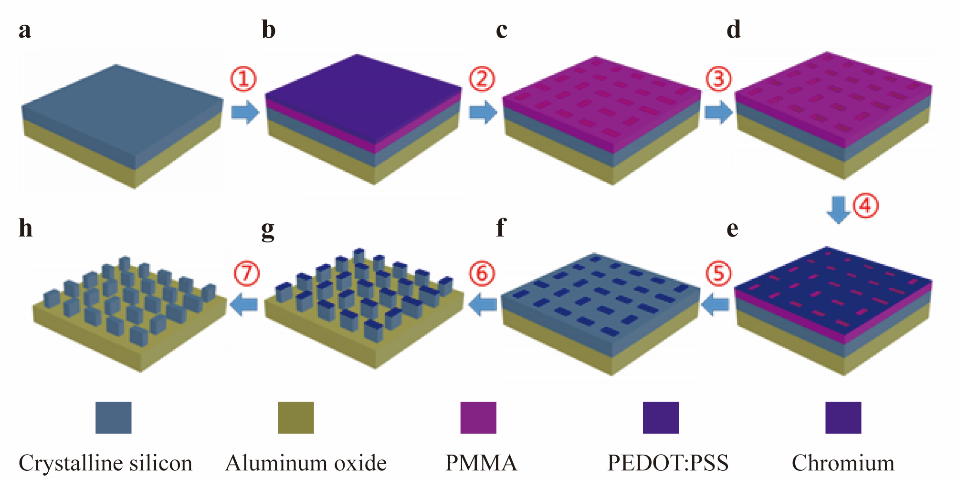


**Fig. S3 Schematic diagram of the preparation process of the metasurface used in the experiment.**

The preparation process for the metasurface target used in the experiment is illustrated in Fig. S3. Initially, a layer of polymethyl methacrylate (PMMA) was applied onto the cleaned and dried SOS material as an electron beam resist and cured at 150 °C. Subsequently, a conductive poly(3,4-ethylenedioxythiophene) polystyrene sulfonate (PEDOT: PSS) layer was evenly spin-coated onto the PMMA layer with a constant speed and homogenization time and cured at 90 °C. In the second step, electron beam lithography (EBL) (Raith 150, 30 kV) was used to produce a mask pattern of the nanobrick structure on the PMMA layer, followed by cleaning the sample with deionized water to remove the conductive layer. In the third step, development and exposure procedures were executed to eliminate the same portion of the PMMA material as the nanobrick structure array, thereby preserving only the region outside of the processed pattern. In the fourth step, a 30 nm thick chromium film was deposited on the sample using a thermal evaporator. In the fifth step, the sample was immersed in a hot acetone solution at 75 °C and cleaned using ultrasonic waves to completely remove the PMMA material covering the sample. In the sixth step, reactive ion etching (RIE) technology was employed to etch the crystalline silicon material. The chromium film provided protection, ensuring the processing pattern was fully transferred to the SOS. Finally, a chromium etching solution was used to remove any remaining chromium film. Following these steps, an array of crystalline silicon nanobricks remained on the sapphire substrate.

## Section 2 Photoelectric performance of FAPbBr_2.4_I_0.6_ and FAPbI_3_ DIP-SPD


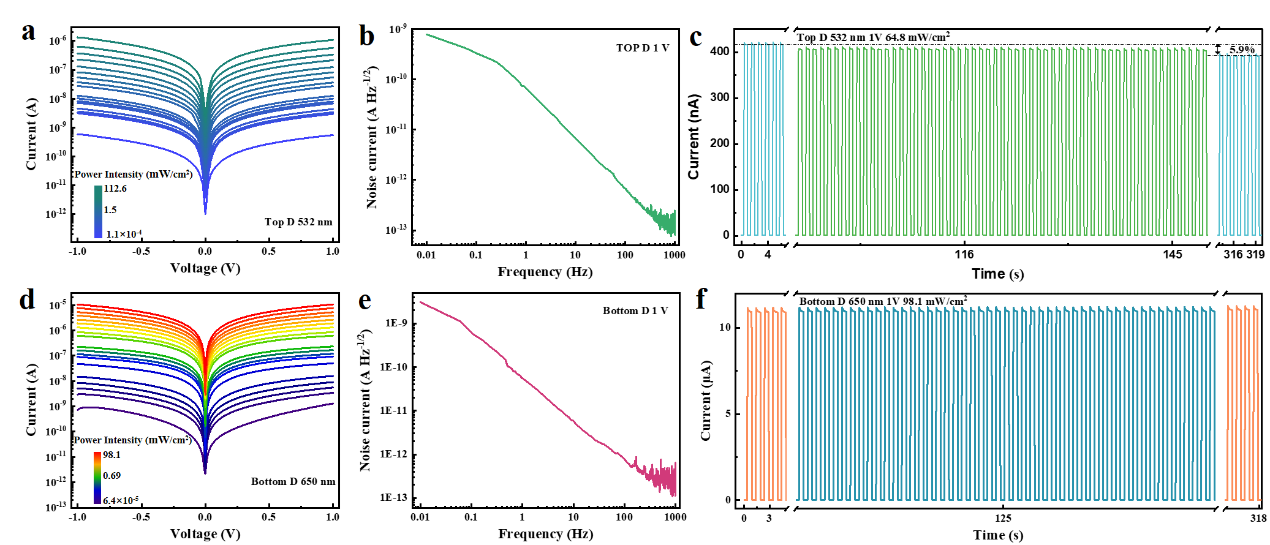


**Fig. S4 Photoelectric performance of FAPbBr_2.4_I_0.6_ and FAPbI_3_ DIP-SPD. a** The photocurrent and dark current of the top FAPbBr_2.4_I_0.6_ device, which is selected under a weak light density from 1.1 × 10^-4^ mWcm^-2^ to 112.6 mWcm^-2^. **b** Frequency-dependent noise current of the top FAPbBr_2.4_I_0.6_ device with 1 V bias. **c** The fast switching response of the top FAPbBr_2.4_I_0.6_ device to light at 1 V bias varies with time and the optical power density of 64.8 mWcm^-2^. **d** The photocurrent and dark current of the bottom FAPbI_3_ device, which is selected under a weak light density from 6.4 × 10^-5^ mWcm^-2^ to 98.1 mWcm^-2^. **e** Frequency-dependent noise current of the bottom FAPbI_3_ device with 1 V bias. **f** The fast switching response of the bottom FAPbI_3_ device to light at 1 V bias varies with time and the optical power density of 98.1 mWcm^-2^.


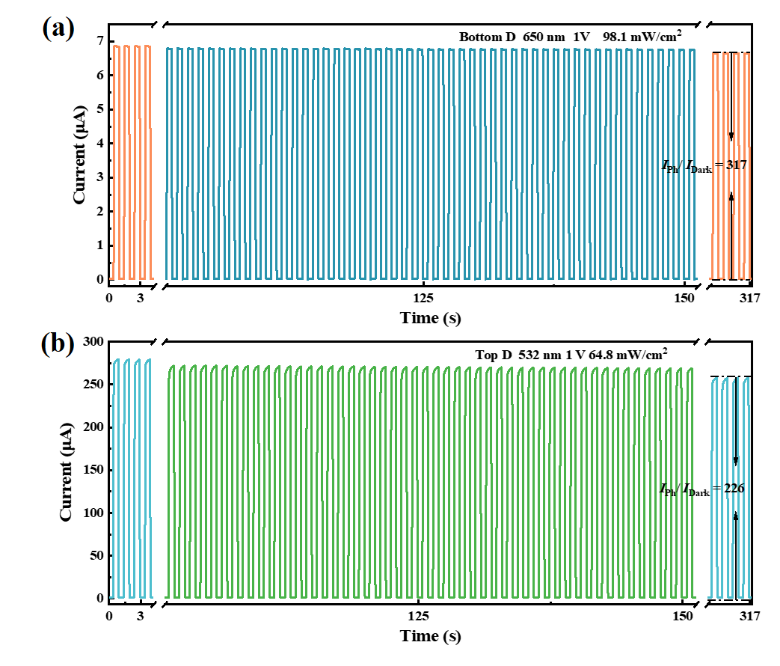


**Fig. S5 The *I-t* curves of the a) FAPbI_3_ and b) FAPbBr_2.4_I_0.6_ devices under switch irradiation, after the devices have been placed for 10 months.**

To further investigate the photoelectric responsiveness of FAPbBr_2.4_I_0.6_ and FAPbI_3_ DIP-SPD, the dark current and the photocurrent response of DIP-SPD under different laser irradiations were tested. The effective detection area of the DIP-SPD can be calculated based on the number and dimensions of interdigital electrode pairs used to output signals. In this case, 20 interdigital electrode pairs with an interdigital spacing of 80 μm and an interdigital length of 3 mm are employed, resulting in an effective detection area of 4.8 × 10^-2^ cm^2^. As displayed in Fig. S4(a), at an applied bias voltage of 1 V, the FAPbBr_2.4_I_0.6_ device exhibits a low dark current of 550 pA. Upon laser irradiation with a spot area of 0.45 cm^2^ and a power density of 112.6 mWcm^-2^, the photocurrent reaches 1.1 μA, with an ON/OFF switching ratio of 2 × 10^3^. The minimum responsive optical power density of the FAPbBr_2.4_I_0.6_ device is as low as 1.1 × 10^-4^ mWcm^-2^. Figure S4(d) presents the photoelectric characteristics of the FAPbI_3_ device. In comparison with the FAPbBr_2.4_I_0.6_ device, the FAPbI_3_ device exhibits an ON/OFF switching ratio of 10^4^ and a minimum detectable optical power of 6.4 × 10^-5^ mWcm^-2^. This suggests that despite the light signal being transmitted through the FAPbBr_2.4_I_0.6_ device, the FAPbI_3_ device was still capable of adequately detecting the signal.

Figures S4(b) and S4(e) present the frequency-dependent noise currents of the FAPbBr_2.4_I_0.6_ and FAPbI_3_ devices, respectively. The noise current follows an inverse proportionality with frequency, leading to a decrease in noise current at higher frequencies, which improves the detection capability of the DIP-SPD for weak signals at high frequencies. Figure S4(c) presents the results of the optical switching cycle stability test of the FAPbBr_2.4_I_0.6_ device under 1 V bias and 64.8 mWcm^-2^ 532 nm laser irradiation. The test indicates that the top FAPbBr_2.4_I_0.6_ device demonstrates a stable long-term switching response, with only a 5.9% reduction in the amplitude of the output photocurrent signal observed within 320 s. Meanwhile, Fig. S4(f) shows that the bottom FAPbI_3_ device under 1 V bias and 98.1 mWcm^-2^ 650 nm laser irradiation exhibits almost unchanged output photocurrent signal amplitude. To further demonstrate the reliability of the devices, Fig. S5 illustrates the *I*-*t* curves of the devices after being stored for 10 months in a nitrogen glove box. The device shows a good shelf life and its performance remains adequate for imaging data acquisition since it can still stabilize the switching even under strong light exposure lasting over 300 seconds. This further illustrates the reliability of the device.

## Section 3 DLI-PD single-pixel imaging system in complex environment

### 3.1 Hadamard patterns utilized in experiments

To enable the single-pixel detector to obtain the spatial information of a target, it is common practice to modulate the target image with a series of mask patterns. The size of the mask pattern (*m* × *n*) determines the dimension of the reconstructed image. Hadamard patterns, which are commonly used in sensing and imaging as a sampling base, are also frequently employed in single-pixel imaging (SPI)^1,2^. Hadamard patterns are generated by the Hadamard matrix, each of which has a pixel value of +1 or -1 and is orthogonal to each other. The order of the Hadamard matrix must satisfy the condition *m* = *n* = 2*^k^* (where *k* is a positive integer), and all higher-order Hadamard matrices can be derived from the original second-order Hadamard matrix $H^{2}$.

$$\begin{aligned} H^{2}=\left[ \begin{matrix} 1 & 1 \\ 1 & -1 \end{matrix} \right],\#(S1) \end{aligned}$$

$$\begin{aligned} H^{2^{k}}=\left[ \begin{matrix} H^{2^{k-1}} & H^{2^{k-1}} \\ H^{2^{k-1}} & -H^{2^{k-1}} \end{matrix} \right]. \#(S2) \end{aligned}$$

Because $H^{2^{k}}$ is an orthogonal matrix, which satisfies $HH^{T}=qI$, where $I$ is a scalar matrix and $q$ is a real constant, the reconstruction of the target image through Hadamard patterns does not require matrix inversion^3^. To generate a series of Hadamard patterns, each row of the Hadamard matrix can be multiplied by each column. This can be expressed as $H_{cr}=H(:,c)\cdot H(r,:)$, where $H(:,c)$ and $H(r,:)$ (1 ≤ *c* ≤ *n*, 1 ≤ *r* ≤ *n*) represent the *c*-th column and *r*-th row of the Hadamard matrix, respectively. Additionally, since DMD is limited to displaying only two elements (0 and 1) instead of the +1 and -1 values in Hadamard patterns, it is necessary to display Hadamard patterns through differences. Although this approach doubles the number of Hadamard patterns required, it also helps to eliminate any offsets in the reconstructed image that may arise from slow changes in the brightness of the background light or the illumination source^4^. Figure S6 displays some of the Hadamard patterns utilized for digital micromirror device (DMD) display.


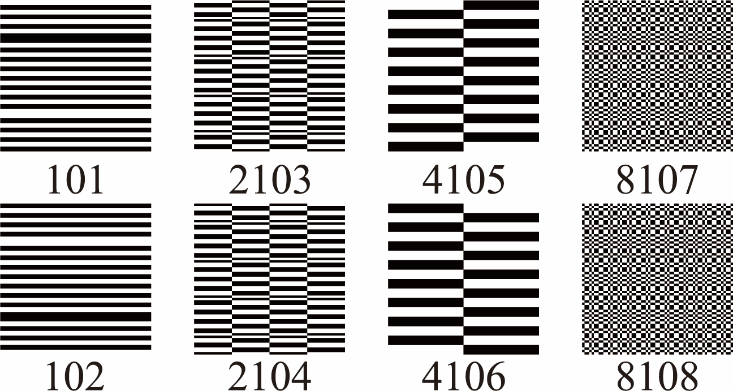


**Fig. S6 Several Hadamard patterns are shown by DMD.**

### 3.2 Simulation analysis of the reconstructed image quality of the bottom FAPbI_3_ layer

To evaluate the effect of the residual signal of the green image on the reconstructed image of the bottom FAPbI_3_ layer, we utilized the peak signal-to-noise ratio (*P_SNR_*) to quantitatively assess the imaging quality of the bottom FAPbI_3_ layer via simulation experiment. The definition of *P_SNR_* is given as follows:

$$\begin{aligned} P_{SNR}=10\log_{10} \frac{M_{max}^{2}}{M_{mse}},\#\left( S3 \right) \end{aligned}$$

where $M_{max}$ is the maximum pixel value of the image, $M_{mse}$ is the mean square error of the reconstructed image, given by

$$\begin{aligned} M_{mse}=\frac{1}{M}\sum_{x}^{M} \left[ R\left( x \right)-I\left( x \right) \right]^{2},\#\left( S4 \right) \end{aligned}$$

where $R\left( x \right)$ and $I\left( x \right)$ are the pixel values of the reconstructed image and the target image, respectively. The simulation result is shown in Fig. S7. In the simulation, we used Fig. S7(b) as the residual signal of the green image and calculated the *P_SNR_* value between the red image (Fig. S7(a)) and the reconstructed images (Figs. S7 (c)-(f)) of the FAPbI_3_ layer. The *P_SNR_* value of the reconstructed image with 10% residual is about 27.60 dB, indicating that the result of the FAPbI_3_ layer has a high signal-to-noise ratio and is not affected by the residual signal of the green image. The *P_SNR_* values of the reconstructed images corresponding to residuals of 30%, 50%, and 70% are approximately 19.25 dB, 15.97 dB, and 14.09 dB, respectively. Only when the residual signal is greater than 30%, the green image will become the prominent background in the reconstructed image. Thus, our detector can be used for high-quality image reconstruction.


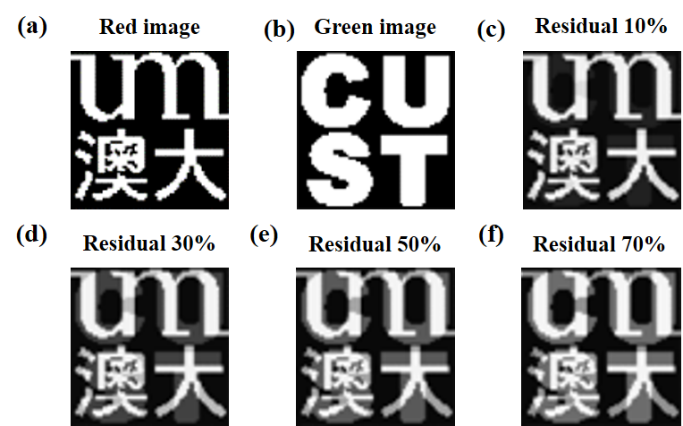


**Fig. S7 Simulation results of FAPbI_3_ layer in DIP-SPD.**

### 3.3 Single-pixel imaging results of DIP-SPD at different DMD modulation frequencies

Figure S8 displays the SPI results of DIP-SPD at different DMD modulation frequencies. Figure S8(a) shows the imaging result obtained using a traditional camera under irradiation with an RCP red laser beam. Figures S8(b) to S8(e) show the reconstructed images obtained using DIP-SPD under irradiation with both RCP red and green laser beams. These images depict the SPI results of DIP-SPD at different DMD modulation frequencies: 1000 Hz in (b), 5000 Hz in (c), 10000 Hz in (d), and 18000 Hz in (e). When using Hadamard patterns to reconstruct a 64 × 64 pixels image, 8192 modulation patterns are required. As a result, the measuring time for the four results from (b) to (e) is 8.192 s, 1.6384 s, 0.8192 s, and 0.4551 s, respectively. The imaging results show that the noise in the reconstructed image of DIP-SPD increases with the DMD modulation frequency, leading to a decrease in imaging quality. However, even at a modulation frequency of 18000 Hz, the target pattern can still be clearly identified.


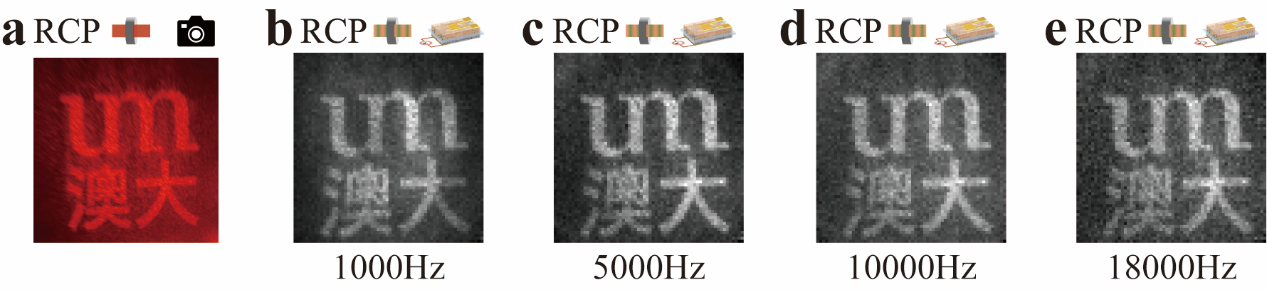


**Fig. S8 Comparison of single-pixel imaging results of DIP-SPD under different DMD modulation frequencies.** **a** Imaging result of the traditional camera under the RCP red lasers. **b** ~ **e** Imaging results of the DIP-SPD at different DMD modulation frequencies (1000Hz, 5000Hz, 10000Hz, and 18000Hz) under illumination with both RCP red and green laser beams.

### 3.4 Imaging results under irradiation with linearly polarized light of mixed wavelengths

Since a beam of light in a linearly polarized state can be decomposed into a left-handed circularly polarized light and a right-handed circularly polarized light. Therefore, when linearly polarized light with mixed wavelengths is incident onto the metasurface, four different images are reconstructed simultaneously and superimposed. When using the conventional camera to capture the metasurface images, the result is shown in Fig. S9(a). The imaging result after adding a scattering medium in front of the conventional camera is shown in Fig. S9(b). Similar to the result when circularly polarized light of mixed wavelengths is incident, the contours of the metasurface images have been completely unrecognizable.


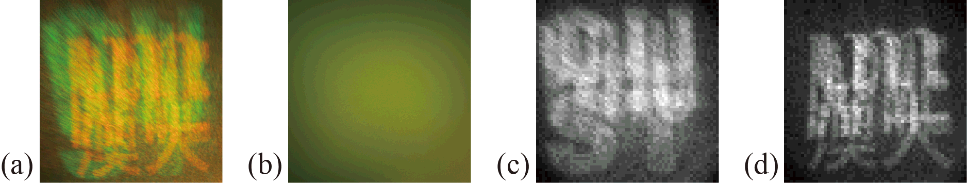


**Fig. S9 Imaging results of the conventional camera and the DIP-SPD when linearly polarized light with mixed wavelengths is incident onto the metasurface. (**a) The imaging result of the commercial camera when linearly polarized light with mixed wavelengths is incident onto the metasurface. (b) The imaging result of the commercial camera when linearly polarized light with mixed wavelengths is incident onto the metasurface in the presence of a scattering medium. (c) and (d) The imaging results corresponding to different colors extracted by the DIP-SPD simultaneously when linearly polarized light with mixed wavelengths is incident onto the metasurface.

When linearly polarized light with mixed wavelengths is incident onto the metasurface, the imaging results of our designed DIP-SPD are shown in Figs. S9(c) and S9(d). Because the DIP-SPD can only identify different wavelengths but not different polarization states, the imaging result of each layer of the DIP-SPD device is a superposition of images corresponding to two different polarizations of the same color. Figs. S9(c) and S9(d) show the reconstructed green and red images of the DIP-SPD device with the linearly polarized incident light, respectively. Because our DIP-SPD is capable of imaging in complex environments, the imaging results of the DIP-SPD in the presence of a scattering medium are similar to Figs. S9(c) and S9(d).

### 3.5 Effect of the scattering medium on traditional camera imaging

Figure S10 displays the microscopic imaging results of the scattering medium under varying magnifications. The images illustrate that the surface of the laminating pouch film contains multiple irregular protrusions, which scatter light and result in blurry imaging using traditional cameras. To better demonstrate the effect of the scattering medium on the imaging performance of traditional cameras, we partially covered only one-half of the metasurface image (the portion within the yellow dotted box) with the medium, while leaving the other half (the portion within the blue dotted box), as illustrated in Fig. S11. Figure S11 depicts that the traditional camera is unable to obtain clear imaging results for the part of the target image shielded by the scattering medium, regardless of the type of laser used as the light source.


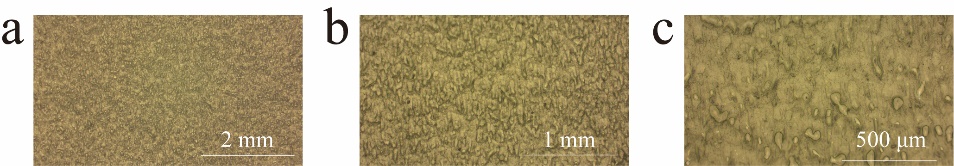


**Fig. S10 Microscopic imaging results of the scattering medium under different magnifications.**


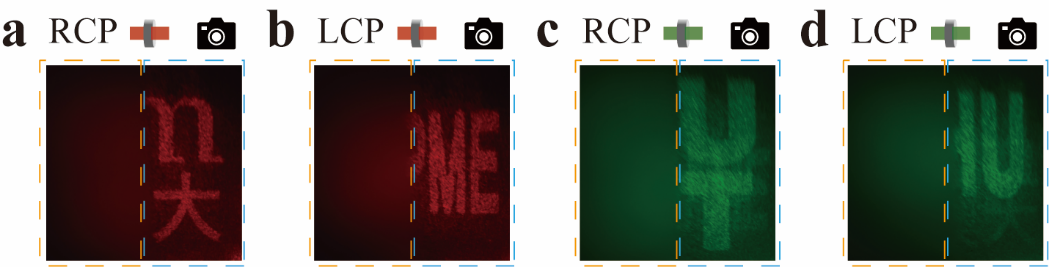


**Fig. S11 Traditional imaging results when half of the metasurface target is obscured by a scattering medium.**

### 3.6 Single-pixel signal in an environment with scattering media

The imaging of a target object by a conventional camera is realized by point-to-point mapping of the target object to each pixel of the imaging chip. If there is a scattering medium in front of a conventional camera, it will change the propagation trajectory of the detected photons, resulting in an error in the point-to-point mapping, and thus the correct image information cannot be captured. Consequently, in the presence of scattering media, conventional cameras are unable to reconstruct a clear image of the target.


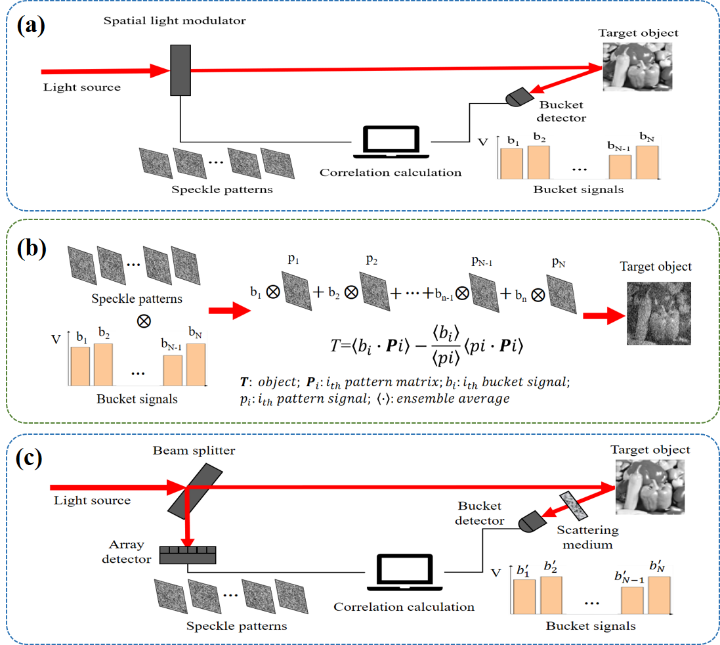


**Fig. S12 Schematic diagram of the principle of single-pixel imaging.** (a) Detection process of single-pixel imaging. (b) Reconstruction process of single-pixel imaging. (c) Detection process of single-pixel imaging with the presence of a scattering medium.

As shown in Fig. S12, unlike the imaging principle of conventional cameras, single-pixel imaging is capable of reconstructing a target image without an array of detectors. In the detection stage (Fig. S12(a)), the target object is modulated by a series of speckle patterns ($P_{i}$), and the corresponding total light intensity signals ($b_{i}$)are acquired using a single-pixel detector with a bucket detection. During image reconstruction (Fig. S12(b)), the recorded light intensity signals are correlated with the modulation patterns to recover the image of the target object (*T*). When a scattering medium is present in front of the single-pixel detector (Fig. S12(c)), the total intensity signal corresponding to the speckle pattern will change to $b_{i}^{'}=k\cdot b_{i}$, where $k$ is the attenuation factor introduced by the scattering medium. Substituting $b_{i}^{'}$ into the reconstruction equation $T\text{=}\left\langle b_{i}\cdot\boldsymbol{P}i \right\rangle-\frac{\left\langle b_{i} \right\rangle}{\left\langle pi \right\rangle}\left\langle pi\cdot\boldsymbol{P}i \right\rangle$, we can obtain $T^{'}\text{=}k\cdot\left( \left\langle b_{i}\cdot\boldsymbol{P}i \right\rangle-\frac{\left\langle b_{i} \right\rangle}{\left\langle pi \right\rangle}\left\langle pi\cdot\boldsymbol{P}i \right\rangle\right)=k\cdot T$. The new reconstructed image $T^{'}$ is simply multiplied by a scale factor, which does not affect the final imaging result. Therefore, although the presence of a scattering medium disrupts the point-to-point mapping relationship required by conventional cameras, as long as the detected photons can pass through the scattering medium and be collected by the single-pixel detector, our method can image the target object without the need to know the a priori knowledge of the scattering medium. However, it needs to be noted that the scattering medium should be in a stable state during our measurements (~1s) to ensure the factor *k* is constant. Otherwise, our single-pixel imaging method will fail for a fast-changing scattering medium.

### 3.7 Single pixel signal in background light environment


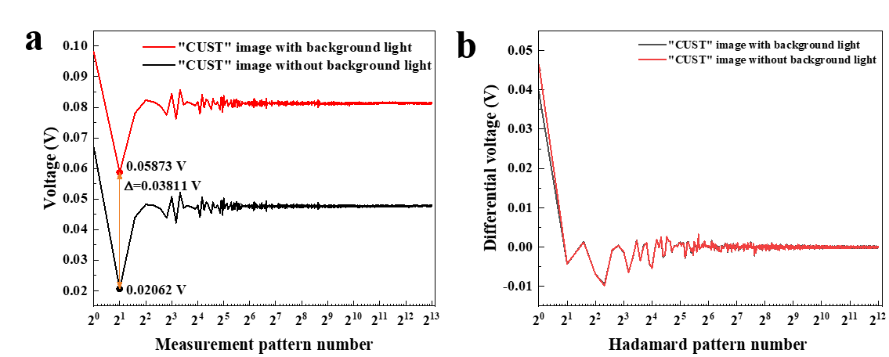


**Fig. S13 Signal amplitude variation of DLI-PD when the backlight is turned on and off.**

Figure S13 presents the intensity variation of the output signal of the top FAPbBr_2.4_I_0.6_ device on DIP-SPD under the illumination of RCP red-green lasers when the background light is turned on and off. Figure S13(a) displays the voltage intensity value of the modulation signal for each measurement pattern. When the background light is turned on, the output signal curve of the device shifts upward as a whole, but the relative change trend remains unchanged. This indicates that the presence of the background light only adds a voltage bias to the output signal of the DIP-SPD. Figure S13(b) shows the modulated signal of Hadamard patterns for target image reconstruction after differential processing. The results presented in Fig. S13(b) indicate that the differential signal intensity used for image reconstruction remains consistent regardless of the presence or absence of the background light. Moreover, the quality of the reconstructed images is almost identical under both conditions, proving the strong background light resistance of the DIP-SPD. Hence, even with the background light turned on, the DIP-SPD can still effectively capture the superimposed dual-color metasurface images with exceptional clarity.

## References

1 Pratt, W. K. *et al.* Hadamard transform image coding. *Proc. IEEE* **57**, 58-68 (1969).

2 Duarte, M. F. *et al.* Single-pixel imaging via compressive sampling. *IEEE Signal Process. Mag.* **25**, 83-91 (2008).

3 Gibson, G. M. *et al.* Single-pixel imaging 12 years on: a review. *Opt. Express* **28**, 28190-28208 (2020).

4 Ye, Z. *et al.* Simultaneous full-color single-pixel imaging and visible watermarking using Hadamard-Bayer illumination patterns. *Opt. Lasers Eng.* **127**, 105955 (2020).
